# Co-Administration of Remdesivir and Azithromycin May Protect against Intensive Care Unit Admission in COVID-19 Pneumonia Requiring Hospitalization: A Real-Life Observational Study

**DOI:** 10.3390/antibiotics11070941

**Published:** 2022-07-14

**Authors:** Andrea Ticinesi, Domenico Tuttolomondo, Antonio Nouvenne, Alberto Parise, Nicoletta Cerundolo, Beatrice Prati, Ilaria Zanichelli, Angela Guerra, Nicola Gaibazzi, Tiziana Meschi

**Affiliations:** 1 Department of Medicine and Surgery, University of Parma, Via Antonio Gramsci 14, 43126 Parma, Italy; domenico.tuttolomondo@unipr.it (D.T.); ilaria.zanichelli@unipr.it (I.Z.); angela.guerra@unipr.it (A.G.); tiziana.meschi@unipr.it (T.M.); 2 Geriatric-Rehabilitation Department, Azienda Ospedaliero-Universitaria di Parma, Via Antonio Gramsci 14, 43126 Parma, Italy; anouvenne@ao.pr.it (A.N.); aparise@ao.pr.it (A.P.); ncerundolo@ao.pr.it (N.C.); bprati@ao.pr.it (B.P.); 3 Department of Cardiology, Azienda Ospedaliero-Universitaria di Parma, Via Antonio Gramsci 14, 43126 Parma, Italy; ngaibazzi@ao.pr.it

**Keywords:** SARS-CoV-2, antiviral, antibiotic, azithromycin, remdesivir

## Abstract

The benefits of remdesivir treatment, with or without co-administration of antibiotics such as azithromycin, are uncertain in COVID-19 pneumonia. The aim of this retrospective single-center study was to assess the effects of remdesivir, with or without azithromycin, on hospital mortality, intensive care unit (ICU) admission, and need of non-invasive ventilation. The clinical records of the COVID-19 patients hospitalized in an Italian ward in March 2021 were analyzed, and data on comorbidities and clinical, radiological, and laboratory presentation of the disease were collected. Among 394 participants (234 M), 173 received remdesivir (43.9%), including 81 with azithromycin (20.5%). Remdesivir recipients were younger, with less comorbidities, and had better PaO_2_/FiO_2_ and clinical outcomes, including reduced mortality, but the differences were not independent of covariates. Rates of ICU transferal were 17%, 9%, and 1% in the no remdesivir, remdesivir without azithromycin, and remdesivir/azithromycin groups, respectively. In a stepwise multivariate logistic regression model, remdesivir/azithromycin co-treatment was independently associated with reduced ICU admission (vs remdesivir alone, OR 0.081, 95% CI 0.008–0.789, *p* = 0.031; vs no remdesivir, OR 0.060, 95% CI 0.007–0.508, *p* = 0.010). These data suggest that the therapeutical effect of remdesivir in COVID-19 pneumonia may be potentiated by azithromycin. The association between the two drugs should be further investigated.

## 1. Introduction

Current guidelines consider intravenous steroid treatment as the mainstay of pharmacological treatment of severe and critical forms of COVID-19 pneumonia, provided that it is not administered in the earliest days of symptoms [1,2]. Steroids, and particularly dexamethasone, have in fact proved effective in reducing mortality and, to a lesser extent, also the duration of mechanical ventilation, if needed [1,2,3]. In selected cases, steroids can be administered in association with IL-6 receptor blockers or baricitinib [1,2].

Antiviral drugs available before emergence of the pandemic have shown little or no efficacy in reducing mortality or mechanical ventilation in severe forms of COVID-19 requiring hospitalization [1,2]. The only exception is represented by remdesivir, whose administration within the first ten days of symptoms to patients with non-critical illness has shown some benefits in terms of need of ventilation and its duration, but no effect on mortality, in comparison with support treatment only [1,2,4,5,6].

However, real-life studies investigating the clinical outcomes of patients receiving intravenous remdesivir treatment during hospitalization for severe COVID-19 pneumonia have produced conflicting results. Some reports have shown little or no impact from this treatment, either alone or in association with steroids, on clinically relevant outcomes such as intensive care unit (ICU) admission or mortality [7,8,9,10]. Conversely, other studies suggested that it could be useful, as an add-on treatment, in adults with persistent fever and dyspnea and moderate lung involvement on chest CT, reducing ICU admission, length of hospital stay, and even mortality [11,12,13,14,15], especially when administered in the first 5 days of symptoms [15].

In the earliest phases of the COVID-19 pandemic, the use of azithromycin has also been proposed as a therapeutical option for its potential antiviral and anti-inflammatory effects shown in vitro [16]. Trials investigating the efficacy of azithromycin alone against severe forms of COVID-19 have produced negative results, and its use is no longer recommended [1,2,17,18]. However, antibiotic treatment is still part of the clinical management of severe COVID-19, especially in patients showing multiple pulmonary infiltrates on chest imaging or marked elevation of serum inflammation indexes suggesting the presence of a high risk of bacterial superinfection [2,19]. In these cases, azithromycin is commonly used for its antibacterial action with low risk of generating resistance.

To date, there is still uncertainty on the clinical effectiveness of the association between remdesivir and other drugs showing in vitro activity against SARS-CoV-2, including azithromycin, in patients with severe COVID-19 pneumonia requiring hospitalization. Furthermore, the real-life clinical benefits of remdesivir are far from fully understood, especially in older patients with multiple comorbidities, who are typically excluded from clinical trials.

The aims of this real-life observational study were to describe the clinical characteristics of patients receiving remdesivir during the third pandemic wave (March 2021) in an Italian hub for the care of COVID-19, and to assess the effects of remdesivir treatment, either with or without azithromycin, on clinical outcomes.

## 2. Materials and Methods

### 2.1. Study Setting, Design, and Inclusion Criteria

This was a retrospective, real-life, observational study conducted in the context of the third pandemic wave in Italy in March 2021, which was mainly sustained by the emergence of the alpha SARS-CoV-2 variant [20]. The setting of the study was the Internal Medicine Unit of the Geriatric-Rehabilitation Department of Parma University-Hospital, which had been identified as the main hub for the hospital care of patients with COVID-19 of the whole Parma Province (>450,000 inhabitants) since the first wave in 2020 [21,22].

Included in the study were all patients admitted 1–31 March 2021 with positive nasopharyngeal swab for SARS-CoV-2, presence of symptoms compatible with COVID-19, and chest Computed Tomography (CT) results that were positive or indeterminate for the presence of COVID-19 pneumonia. Excluded were patients who did not undergo chest imaging, patients with missing data on treatments against COVID-19 administered before and during hospital stay, and patients who explicitly denied their consent for study inclusion and data treatment.

### 2.2. Procedures and Data Collection

The clinical records and discharge forms of all eligible patients were reviewed by trained staff members. Data on the clinical presentation of COVID-19 (type and duration of symptoms, vital signs, chest CT findings, routine lab tests, and arterial blood gas analysis), number and type of comorbidities, and number of chronic medications were collected on case report forms. Namely, the extension of lung abnormalities on CT was measured through visual scoring, expressed as percentage of the lung parenchyma with ground-glass pattern or consolidations [23]. Respiratory function was assessed through calculation of the ratio between arterial oxygen partial pressure on arterial blood gas analysis and fraction of inspired oxygen on admission (PaO_2_/FiO_2_). Lab tests included serum levels of the main inflammatory markers upon admission: C-reactive protein (CRP), procalcitonin (PCT), and IL-6. The severity of COVID-19 was also classified according to the WHO COVID-19 Scale [24,25].

Treatments administered against COVID-19 (steroids, IL-6 receptor antagonists, remdesivir, antibiotics including azithromycin) were also collected by review of clinical records, along with the maximal oxygen or ventilatory need during hospital stay. The hospitalization outcome (discharge vs death) was also assessed.

### 2.3. Exposure and Outcome Variables

Patients included in the study were treated in accordance with the recommendations for COVID-19 in force in Italy in March 2021 [26]. Allocation to treatments (corticosteroids, enoxaparin, IL-6 antagonists, remdesivir, antibiotics including azithromycin) was independent of the study. Treatment with remdesivir, either in association with azithromycin or not, was considered the main exposure variable for analyses.

The decision to treat patients with remdesivir was based on the compliance with criteria of prescription issued by the European Medicines Agency and by the Italian Drug Agency (AIFA—Agenzia Italiana del Farmaco) [27] and on the clinical judgement of the prescribing physician. According to AIFA criteria, remdesivir could be prescribed to hospitalized patients with COVID-19 only in case all of the following were satisfied:

- duration of COVID-19 related symptoms not exceeding 10 days.
- presence of lung parenchyma abnormalities on chest imaging.
- no need of oxygen therapy with high-flow nasal cannulae (HFNC), non-invasive or invasive mechanical ventilation, or extracorporeal membrane oxygenation (ECMO).
- serum aspartate and alanine aminotransferase levels not exceeding 5 times the upper limit of reference.
- glomerular filtration rate (GFR) not inferior to 30 mL/min.

Remdesivir was administered intravenously with a load dose of 200 mg on day 1, followed by maintenance doses of 100 mg daily from day 2 to day 5.

The decision to treat patients with azithromycin relied on clinical judgement and was based on the risk of bacterial superinfection of COVID-19-related pulmonary lesions after review of the clinical presentation, chest imaging, and lab tests, including CRP and PCT levels. Azithromycin was administered orally at 500 mg per day for three to five days, according to clinical judgement.

Hospital mortality was considered the main study endpoint. ICU admission, need of invasive or non-invasive mechanical ventilation, and length of overall hospital stay were also considered as secondary endpoints.

### 2.4. Statistical Analyses

Data were expressed as percentages or median and interquartile range (IQR), as appropriate. The clinical characteristics and outcomes were compared after categorizing participants for treatment with remdesivir, and with remdesivir plus azithromycin. When comparing continuous variables, the Mann–Whitney and Kruskal–Wallis tests were used. Significant values were adjusted for potential confounders with the Quade non-parametric Ancova test. Chi-square test and binary logistic regression were used for comparing discrete variables.

Stepwise logistic regression models were used to verify the associations between exposures (particularly treatment with remdesivir and remdesivir/azithromycin association) with primary or secondary outcomes. Age, sex, chest CT visual score, PaO_2_/FiO_2_ on admission, and the number of chronic comorbidities were considered as covariates.

Statistical analyses were performed with the SPSS package (v.28, IBM, Armonk, NY, USA); *p* values were considered significant when <0.05.

## 3. Results

### 3.1. General Characteristics of the Population

In March 2021, 467 patients with COVID-19 were admitted to the study unit. After checking for inclusion and exclusion criteria, 394 patients (234 M, 160 F) were finally included. Among them, 173 patients (43.9%) received remdesivir during hospital stay. Azithromycin was co-administered in 81 remdesivir recipients (20.5% of the whole population) (Supplementary Figure S1).

A comparison between the baseline clinical characteristics of patients who received remdesivir and those who did not is shown in Table 1. Namely, remdesivir recipients were younger (age median 60, IQR 52–71, vs. 71, IQR 61–80 years old, *p* < 0.001), had a reduced burden of comorbidities (median number of chronic illnesses 1, IQR 0–3, vs. 3, IQR 1–4, *p* < 0.001), and better respiratory exchanges (PaO_2_/FiO_2_ median 312, IQR 264–352, vs. 276, IQR 202–328 mmHg, *p* < 0.001). Serum inflammatory markers and neutrophil count were also lower in remdesivir recipients upon hospital admission.

Table 2 shows multiple comparisons of baseline clinical characteristics, after splitting the group of remdesivir recipients between those who received remdesivir alone and those who received remdesivir plus azithromycin. These two groups were comparable for demographic, clinical, and laboratory characteristics.

### 3.2. Effects of Treatments on Mortality

Eighty patients (20.3%) died during hospital stay, 64 (29%) in the group not receiving remdesivir and 16 (9%) in the remdesivir recipients. This difference was not statistically significant after adjustment for age, sex, and parameters of disease severity (Table 3). In the remdesivir group, mortality was not different between those who received azithromycin and those who did not (Table 4).

In a stepwise multivariable logistic regression model, the only factors significantly associated with mortality were age (OR 1.109, 95% CI 1.069–1.150, *p* < 0.001), number of chronic illnesses (OR 1.457, 95% CI 1.210–1.755, *p* < 0.001), chest CT visual score of lung parenchyma involvement (OR 1.027, 95% CI 1.009–1.046, *p* = 0.004), and PaO_2_/FiO_2_ on admission (OR 0.991, 95% CI 0.987–0.995, *p* < 0.001). Neither remdesivir nor azithromycin treatment were independently associated with mortality.

### 3.3. Effects of Treatments on Other Clinical Outcomes

Patients who received remdesivir during hospital stay experienced lower frequency of non-invasive mechanical ventilation (18% vs. 34%) and ICU admission (5% vs. 17%). They also had shorter duration of hospitalization (median 12, IQR 8–19, vs. 14, IQR 10–24 days). However, the differences were not statistically significant after adjustment for age, sex, and parameters of disease severity, except for ICU admission (Table 3). Similar results were obtained when comparing patients receiving remdesivir plus azithromycin and those not on remdesivir treatment (Table 4). The rate of ICU admission was significantly lower, and very low in absolute terms, in those who were treated with remdesivir plus azithromycin (1% vs. 9%, *p* < 0.001).

In a stepwise logistic regression model, accounting also for age, sex, number of chronic illnesses, chest CT visual score of lung parenchyma involvement, and PaO_2_/FiO_2_ on admission, the association of remdesivir plus azithromycin, but not remdesivir alone, was protective against ICU admission (Table 5).

### 3.4. Safety Issues

Treatment with remdesivir was stopped in only three patients, due to onset of bradycardia in two cases and marked aminotransferase elevation in one case. No serious adverse events were reported in clinical records of all other remdesivir recipients included in the study.

## 4. Discussion

In a group of patients hospitalized with severe COVID-19 during the third pandemic wave in Italy, treatment with remdesivir, administered in compliance with the recommendations from regulatory authorities, was associated with reduced mortality and reduced need of escalating ventilatory support. However, these outcomes were not independent of the baseline clinical presentation of COVID-19 and demographical characteristic, which were substantially different between remdesivir recipients and other patients. The co-administration of remdesivir and azithromycin, instead, seemed associated with reduced need of ICU admission independently of covariates.

To the best of our knowledge, this is the first study specifically investigating the effect of the co-administration of remdesivir with azithromycin in the scientific literature on COVID-19.

Treatment with remdesivir emerged as a promising therapeutical option for COVID-19 in the second half of 2020, with evidence that it could reduce the recovery time and length of hospital stay, though not affecting mortality [28]. Systematic reviews and meta-analyses conducted on further randomized controlled trials substantially confirmed that, in patients without illness requiring ventilatory support, the administration of remdesivir is associated with some clinical benefit, with reduced need of escalating ventilatory support, but has no apparent effect on mortality [4,5,6]. The severity of clinical presentation of COVID-19 patients during the second and third pandemic wave in 2020–2021, before the completion of mass vaccination campaigns, and the absence of specific and effective drugs against SARS-CoV-2, have made remdesivir administration particularly frequent in the hospital setting, despite the absence of clear advantages against mortality. To date, real-life studies have provided conflicting results, with some reports suggesting clear benefits from remdesivir administration, and other yielding negative results [7,8,9,10,11,12,13,14,15].

Timing of remdesivir administration may be a critical point in this regard. When administered within five days from the symptom onset, remdesivir may be associated with reduced rate of clinical progression towards severe respiratory failure, in comparison with administration from day 5 to day 10 of symptom onset [29,30]. A recent randomized controlled trial suggests that precocious use of remdesivir in the pre-hospital setting is associated with dramatically lower rates of hospitalization and progression towards severe COVID-19 forms [31]. However, this drug requires intravenous administration, which is not always feasible in the community setting, especially in the context of a pandemic. Notably, in our study, the outcomes of remdesivir were unrelated with the timing of its administration.

Few reports have evaluated possible favorable interactions between remdesivir and other drugs used in patients with COVID-19. However, in clinical practice, pharmacologic treatment of SARS-CoV-2 pneumonia is rarely based on a single drug, in contrast with the design of most clinical trials. Most of the patients included in our investigation received also corticosteroids and antibiotics, in addition to remdesivir. This circumstance could contribute to explain why remdesivir administration was not independently associated with clinical benefits, because its effect was probably masked by steroid treatment. On the other side, the reduced need of ICU admission in patients who were treated with both remdesivir and azithromycin suggests that the clinical benefits of remdesivir could be reinforced by the administration of other drugs exhibiting anti-SARS-CoV-2 activity in vitro, like azithromycin. Also, it may contribute to explain why real-life studies on remdesivir efficacy have produced conflicting results [7,8,9,10,11,12,13,14,15].

Azithromycin has known antiviral effects in vitro that have been demonstrated also against SARS-CoV-2 [32]. Its efficacy as stand-alone treatment against COVID-19 is negligible [17,18], and in the early phases of the pandemic, it was studied as a therapeutic option in association with hydroxychloroquine, with a paradoxical increase of mortality [33]. Our findings, instead, suggest that the interaction between remdesivir and azithromycin should be further studied, both at the molecular and clinical level, in order to optimize recommendations for the hospital management of severe COVID-19.

Our study also highlighted that, in clinical practice, remdesivir was mainly administered to patients below the age of 70, with low comorbidity burden, moderate lung abnormality extension on chest imaging, and fairly good respiratory involvement on hospital admission. These patients may have lower risk of adverse outcomes, independently of treatments administered during hospital stay. In fact, the dramatic differences in outcomes between remdesivir recipients and other patients disappeared after correction for age and parameters associated with disease severity on admission, such as the PaO_2_/FiO_2_ ratio. These data reinforce the importance of evaluating the PaO_2_/FiO_2_ ratio on admission of patients with COVID-19, because it may represent the single parameter with the highest outcome prediction capacity [34].

However, the majority of severe cases of COVID-19 generally involve older subjects, with several comorbidities and relevant pneumonia extension on chest imaging [22,34,35]. Age-related frailty represents a consistent risk factor for adverse outcomes in COVID-19 [35,36], also for the increased risk of bacterial superinfection [37]. Many older subjects included in our study were excluded from remdesivir treatment because their clinical presentation on admission was not compatible with the criteria of prescription [27]. The role of frailty as modifier of the efficacy of anti-COVID-19 treatments, including remdesivir, should be studied in the future.

Our study has some limitations that should be carefully considered in result interpretation. First, the retrospective design is not ideal for assessing outcomes of pharmacologic treatments, and selection bias could not be excluded. In fact, the presence of strict regulatory criteria for prescription of remdesivir in Italy [27], which were followed in patients included in the study, may have dramatically affected allocation to remdesivir treatment. This is the main reason why demographic and clinical differences between participants who were treated with remdesivir and those who were not were relevant. Thus, statistical correction for the main parameters may not have adequately accounted for these differences, making the two groups not fully comparable. Similarly, in patients who required antibiotic therapy, azithromycin was prescribed mainly based on clinical judgement and in case of moderate respiratory involvement, while patients with severe or critical illness received other broad-spectrum antibiotics. This circumstance may have also affected allocation to azithromycin and should be regarded as a possible source of bias.

Furthermore, the sample size was not sufficiently large to explore clinical benefits of remdesivir treatment on secondary outcomes. The study data also refer to a period of predominance of the SARS-CoV-2 alpha variant, preceding mass vaccination against SARS-CoV-2. Vaccination campaigns and the emergence of novel SARS-CoV-2 variants with increased transmission and different clinical presentation have substantially modified the characteristics of patients hospitalized with COVID-19 [38], making the results of the present investigation not automatically transferrable to the current epidemiological situation.

In spite of these limitations, our results could be preliminary to the design of studies investigating the effects of the combination of remdesivir treatment with other drugs on the clinical course of COVID-19 pneumonia, and could serve as hypothesis generators for a better comprehension of the in vivo effects of remdesivir.

## 5. Conclusions

In a real-life setting during the third pandemic wave in Italy, the administration of remdesivir was not independently associated with improved mortality and reduced need of ventilatory support in patients hospitalized with severe COVID-19. However, the co-administration of remdesivir and azithromycin was associated with reduced risk of ICU admission, independently of covariates. The clinical efficacy of the association between remdesivir and azithromycin in COVID-19 should be further investigated.

## Figures and Tables

**Table 1:** Comparison of the main characteristics of patients treated with remdesivir and patients who did not receive the drug.

	No Remdesivir (N = 221)	Treated with Remdesivir (N = 173)	*p*
**Demography and personal history**			
Age, years	71 (61–80)	60 (52–71)	**<0.001**
Female sex, %	42	39	0.641
Chronic comorbidities, number	3 (1–4)	1 (0–3)	**<0.001**
Chronic medications, number	2 (1–5)	1 (0–3)	**<0.001**
Hypertension, %	59	45	**0.005**
Ischemic heart disease, %	15	5	**0.001**
Diabetes, %	21	13	**0.040**
Obesity, %	13	17	0.194
Dyslipidemia, %	21	17	0.309
Atrial Fibrillation, %	16	6	**0.001**
**Chest CT presentation**			
Ground glass abnormalities on CT, %	90	99	**<0.001**
Consolidations on CT, %	69	71	0.782
Visual score of pneumonia extension, %	25 (15–45)	20 (15–35)	0.074
**Clinical presentation upon admission**			
PaO_2_/FiO_2_, mmHg	276 (202–328)	312 (264–352)	**<0.001**
Duration of symptoms, days	6 (2–10)	7 (4–9)	0.150
Fever, %	76	81	0.286
Cough, %	44	53	0.082
Dyspnea,%	58	43	**0.003**
Diarrhoea, %	17	16	0.790
WHO COVID-19 Scale mild, %	10	1	**<0.001**
WHO COVID-19 Scale moderate, %	24	32	0.082
WHO COVID-19 Scale severe, %	27	42	**0.002**
WHO COVID-19 Scale critical, %	38	24	**0.003**
**Blood tests**			
Hemoglobin, g/dL	13.8 (12.2–15.0)	14.0 (12.9–15.0)	0.055
Platelet count, 1000/mm^3^	195 (146–266)	187 (149–223)	0.125
White Blood Cell count, n/mm^3^	6980 (5113–8885)	5500 (4060–7850)	**<0.001**
Neutrophil count, n/mm^3^	5403 (3610–7392)	4400 (2781–6243)	**<0.001**
Creatinine, mg/dL	0.9 (0.7–1.2)	0.8 (0.7–1.0)	**0.004**
C-Reactive Protein, mg/L	62 (29–107)	46 (23–81)	**0.005**
Procalcitonin, ng/mL	0.11 (0.06–0.39)	0.07 (0.04–0.14)	**<0.001**
Interleukin-6, pg/mL	86 (29–182)	79 (34–145)	0.908
D-dimer, ng/mL	940 (532–1635)	608 (420–906)	**<0.001**
**Other treatments administered**			
Enoxaparin, %	95	97	0.401
Corticosteroids,%	90	99	**<0.001**
Antibiotics, %	88	87	0.750
Azithromycin, %	29	47	**<0.001**

Data are shown as median and interquartile range or percentages. Crude comparisons were made with Mann–Whitney test or chi-square test, as appropriate; *p* values < 0.05 are indicated in bold.

**Table 2:** Comparison of the main characteristics of patients who were not treated with remdesivir (1), who received remdesivir but not azithromycin (2), and who received remdesivir plus azithromycin (3).

	No Remdesivir N = 221 (1)	Treated with Remdesivir, No Azithromycin N = 92 (2)	Treated with Remdesivir and Azithromycin N = 81 (3)	*p*	
**Demography and personal history**					
Age, years	71 (61–80)	59 (52–70)	60 (51–72)	**<0.001**	(1) vs. (2) vs. (3)
Female sex, %	42	36	43	0.555	
Chronic comorbidities, number	3 (1–4)	1 (1–3)	1 (0–3)	**<0.001**	(1) vs. (2) vs. (3)
Chronic medications, number	2 (1–5)	1 (0–3)	1 (0–2)	**<0.001**	(1) vs. (2) vs. (3)
Hypertension, %	59	49	41	**0.012**	(1) vs. (3)
Ischemic heart disease, %	15	7	4	**0.009**	(1) vs. (2) vs. (3)
Diabetes, %	21	15	11	0.100	
Obesity, %	13	20	15	0.297	
Dyslipidemia, %	21	15	19	0.515	
Atrial fibrillation, %	16	8	4	**0.008**	(1) vs. (2) vs. (3)
**Chest CT presentation**					
Ground glass abnormalities on CT, %	90	99	99	**0.011**	(1) vs. (2) vs. (3)
Consolidations on CT, %	69	70	72	0.922	
Visual score of pneumonia extension, %	25 (15–45)	20 (15–35)	20 (15–30)	0.153	
**Clinical presentation upon admission**					
PaO_2_/FiO_2_, mmHg	276 (202–328)	319 (273–354)	305 (262–348)	**<0.001**	(1) vs. (2) vs. (3)
Duration of symptoms, days	6 (2–10)	7 (4–9)	6 (4–9)	0.352	
Fever, %	76	80	81	0.559	
Cough, %	44	55	51	0.181	
Dyspnea,%	58	52	32	**<0.001**	(3) vs. (1) vs. (2)
Anosmia, %	7	8	14	0.171	
Diarrhoea, %	17	18	14	0.668	
WHO COVID-19 Scale mild, %	10	1	1	**0.011**	(1) vs. (2) vs. (3)
WHO COVID-19 Scale moderate, %	24	30	35	0.185	
WHO COVID-19 Scale severe, %	27	46	38	**0.005**	(2) vs. (1)
WHO COVID-19 Scale critical, %	38	23	26	**0.011**	(1) vs. (2) vs. (3)
**Blood tests**					
Hemoglobin, g/dL	13.8 (12.2–15.0)	14.0 (12.9–14.9)	14.0 (12.9–15.0)	0.155	
Platelet count, 1000/mm^3^	195 (146–266)	189 (152–224)	185 (148–222)	0.302	
White Blood Cell count, n/mm^3^	6980 (5113–8885)	5930 (4380–8620)	5415 (3758–7318)	**<0.001**	(1) vs. (2) vs. (3)
Neutrophil count, n/mm^3^	5403 (3610–7392)	4574 (3223–6368)	3915 (2526–5822)	**<0.001**	(1) vs. (2) vs. (3)
Creatinine, mg/dL	0.9 (0.7–1.2)	0.9 (0.7–1.0)	0.8 (0.7–1.0)	**0.009**	(1) vs. (3)
C-reactive protein, mg/L	62 (29–107)	47 (23–81)	45 (24–86)	**0.018**	
Procalcitonin, ng/mL	0.11 (0.06–0.39)	0.06 (0.04–0.13)	0.08 (0.05–0.14)	**<0.001**	(1) vs. (2) vs. (3)
Interleukin-6, pg/mL	86 (29–182)	81 (32–155)	73 (41–140)	0.931	
D-dimer, ng/mL	940 (532–1635)	577 (381–909)	628 (441–895)	**<0.001**	(1) vs. (2) vs. (3)
**Other treatments administered**					
Enoxaparin, %	95	98	96	0.623	
Corticosteroids,%	90	99	99	**0.011**	(1) vs. (2) vs. (3)
Antibiotics, %	88	75	100	**<0.001**	(1) vs. (2) vs. (3); (2) vs. (3)

Data are shown as median and interquartile range or percentages. *p* calculated with Kruskal–Wallis test (significance values adapted on Bonferroni correction for multiple testing) or binary logistic regression; *p* values < 0.05 are indicated in bold.

**Table 3:** Comparison of study outcomes between patients who received remdesivir during hospital stay and those who did not.

	No Remdesivir (N = 221)	Treated with Remdesivir (N = 173)	*p* (Unadjusted)	*p* Adjusted for Age, Sex	*p* Adjusted (Model 1)	*p* Adjusted (Model 2)	*p* Adjusted (Model 3)	*p* Adjusted (Model 4)
Hospital death, %	29	9	**<0.001**	**0.005**	**0.016**	0.054	0.409	0.555
NIV, %	34	18	**<0.001**	**<0.001**	**0.001**	0.054	0.095	0.290
ICU admission, %	17	5	**<0.001**	**<0.001**	**<0.001**	**0.004**	**0.011**	**0.026**
Invasive ventilation, %	7	2	**0.027**	**0.019**	**0.022**	0.071	0.176	0.191
Length of stay, days	14 (10–24)	12 (8–19)	**0.027**	0.197	0.336	0.795	0.294	0.417

Model 1 adjusted for age, sex, and number of chronic illnesses. Model 2 adjusted for age, sex, number of chronic illnesses, and chest CT visual score. Model 3 adjusted for age, sex, number of chronic illnesses, and PaO_2_/FiO_2_ on admission. Model 4 adjusted for age, sex, number of chronic illnesses, chest CT visual score, and PaO_2_/FiO_2_ on admission. Value for *p* calculated with binary logistic regression, except for length of stay (unadjusted values calculated with Mann–Whitney test, adjusted values calculated with Quade nonparametric Ancova test; *p* values < 0.05 are indicated in bold. NIV = Non-Invasive Ventilation; ICU = Intensive Care Unit.

**Table 4:** Comparison of study outcomes between patients who received remdesivir azithromycin during hospital stay and those who did not receive remdesivir.

	No Remdesivir (N = 221)	Remdesivir Plus Azithromycin (N = 81)	*p* (Unadjusted)	*p* Adjusted for Age, Sex	*p* Adjusted (Model 1)	*p* Adjusted (Model 2)	*p* Adjusted (Model 3)	*p* Adjusted (Model 4)
Hospital death, %	29	9	**<0.001**	**0.026**	0.070	0.223	0.232	0.445
NIV, %	34	15	**0.001**	**0.001**	**0.002**	**0.031**	**0.028**	0.117
ICU admission, %	17	1	**<0.001**	**0.003**	**0.003**	**0.009**	**0.006**	**0.009**
Length of stay, days	14 (10–24)	12 (9–19)	**0.043**	0.606	0.645	0.869	0.902	0.948

Model 1 adjusted for age, sex, and number of chronic illnesses. Model 2 adjusted for age, sex, number of chronic illnesses, and chest CT visual score. Model 3 adjusted for age, sex, number of chronic illnesses, and PaO_2_/FiO_2_ on admission. Model 4 adjusted for age, sex, number of chronic illnesses, chest CT visual score, and PaO_2_/FiO_2_ on admission. Value of *p* calculated with binary logistic regression, except for length of stay (unadjusted values calculated with Mann–Whitney test, adjusted values calculated with Quade nonparametric Ancova test; *p* values < 0.05 are indicated in bold. NIV = Non-Invasive Ventilation; ICU = Intensive Care Unit.

**Table 5:** Factors independently associated with ICU admission in the studied population on a stepwise logistic regression model.

	Odds Ratio	95% Confidence Interval	*p*
Age, years	0.961	0.933–0.989	**0.007**
Female sex (vs. male)	0.426	0.182–0.977	**0.049**
PaO_2_/FiO_2_, mmHg	0.982	0.977–0.987	**<0.001**
Treatments			**0.034**
Association between remdesivir and azithromycin (vs. no remdesivir)	0.060	0.007–0.508	**0.010**
Association between remdesivir and azithromycin (vs. remdesivir alone)	0.081	0.008–0.789	**0.031**
Remdesivir (vs. no remdesivir)	0.743	0.273–2.023	0.560

Model also accounting for age, sex, number of chronic illnesses, chest CT visual score of lung parenchyma involvement, and PaO_2_/FiO_2_ on admission; *p* values < 0.05 are indicated in bold.

## Data Availability

Access to data, in anonymous form, can be obtained upon reasonable and motivated request to the corresponding author. The subject entitled for data control and management is the Parma University-Hospital (Azienda Ospedaliero-Universitaria di Parma).

## Supplementary Materials

The following supporting information can be downloaded at: https://www.mdpi.com/article/10.3390/antibiotics11070941/s1, Supplementary Figure S1–Modified CONSORT diagram of the study.
