# Supplementary figures and images for: Co-Administration of Remdesivir and Azithromycin May Protect against Intensive Care Unit Admission in COVID-19 Pneumonia Requiring Hospitalization: A Real-Life Observational Study
Source: Antibiotics (Basel). 2022 Jul 14;11(7):941. doi: 10.3390/antibiotics11070941 (PMC9311950; doi:10.3390/antibiotics11070941)

## Supplementary Figure S1

### Modified CONSORT 2010 Flow Diagram of the Study

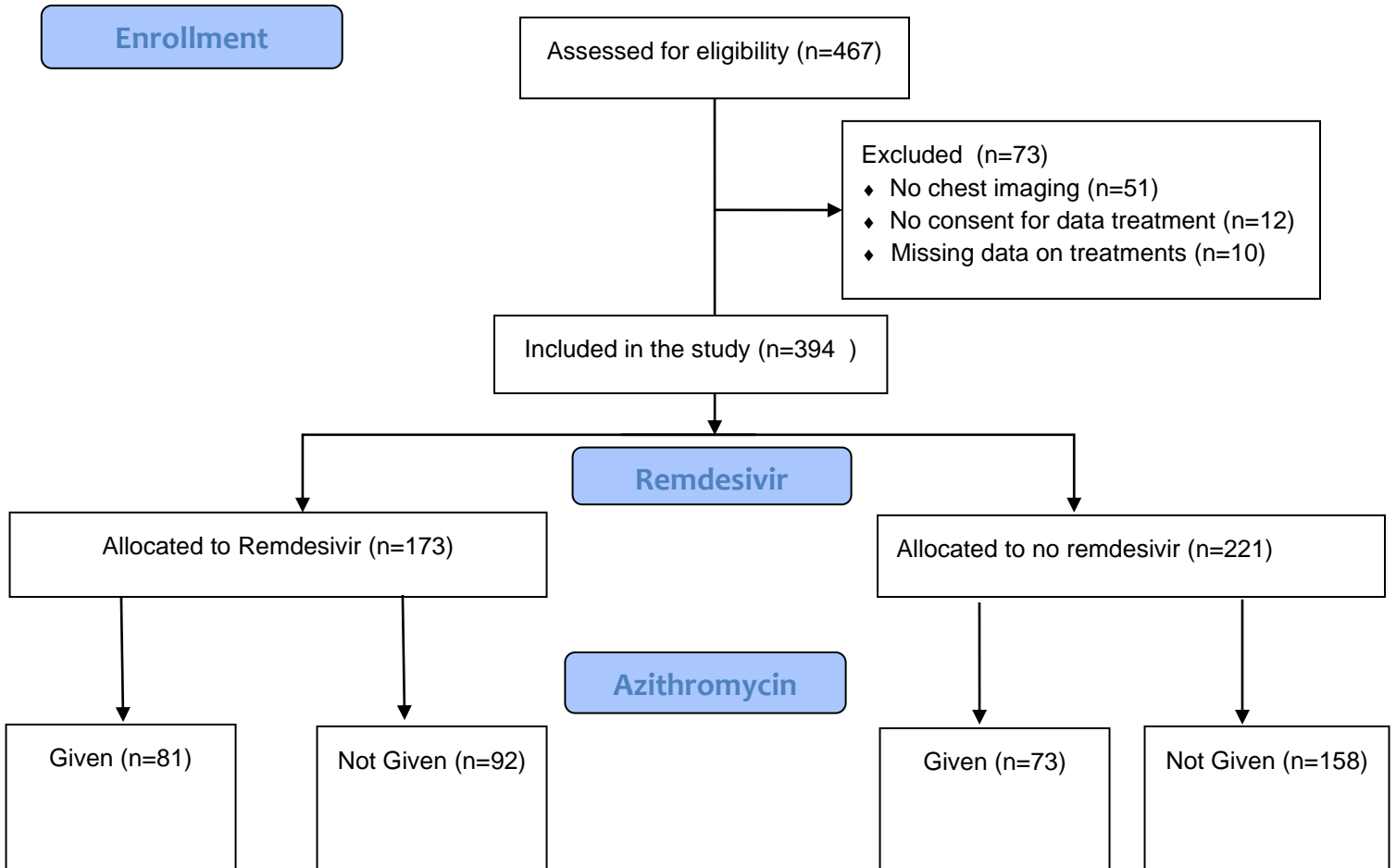

Supplement: Supplementary file 1 [file antibiotics-11-00941-s001.zip › Supplementary Figure S1.pdf]
